# Sleep Quality and Emotional Correlates in Taiwanese Coronary Artery Bypass Graft Patients 1 Week and 1 Month after Hospital Discharge: A Repeated Descriptive Correlational Study

**DOI:** 10.1371/journal.pone.0136431

**Published:** 2015-08-20

**Authors:** Pei-Lin Yang, Guey-Shiun Huang, Chien-Sung Tsai, Meei-Fang Lou

**Affiliations:** 1 School of Nursing, National Defense Medical Center, Taipei, Taiwan; 2 School of Nursing, College of Medicine, National Taiwan University, Taipei, Taiwan; 3 Tri-Service General Hospital, National Defense Medical Center, Taipei, Taiwan; 4 School of Medicine, National Defense Medical Center, Taipei, Taiwan; University of Utah, UNITED STATES

## Abstract

**Background:**

Poor sleep quality is a common health problem for coronary artery bypass graft patients, however few studies have evaluated sleep quality during the period immediately following hospital discharge.

**Purpose:**

The aim of this study was to investigate changes in sleep quality and emotional correlates in coronary artery bypass graft patients in Taiwan at 1 week and 1 month after hospital discharge.

**Methods:**

We used a descriptive correlational design for this study. One week after discharge, 87 patients who had undergone coronary artery bypass surgery completed two structured questionnaires: the Pittsburgh Sleep Quality Index and the Hospital Anxiety and Depression Scale. Three weeks later (1 month after discharge) the patients completed the surveys again. Pearson correlations, t-tests, ANOVA and linear multiple regression analysis were used to analyze the data.

**Results:**

A majority of the participants had poor sleep quality at 1 week (82.8%) and 1 month (66.7%) post-hospitalization, based on the global score of the Pittsburgh Sleep Quality Index. Despite poor sleep quality at both time-points the sleep quality at 1 month was significantly better than at 1-week post hospitalization. Poorer sleep quality correlated with older age, poorer heart function, anxiety and depression. The majority of participants had normal levels of anxiety at 1 week (69.0%) and 1 month (88.5%) as measured by the Hospital Anxiety and Depression Scale. However, some level of depression was seen at 1 week (78.1%) and 1 month (59.7%). Depression was a significant predictor of sleep quality at 1 week; at 1 month after hospital discharge both anxiety and depression were significant predictors of sleep quality.

**Conclusion:**

Sleep quality, anxiety and depression all significantly improved 1 month after hospital discharge. However, more than half of the participants continued to have poor sleep quality and some level of depression. Health care personnel should be encouraged to assess sleep and emotional status in patients after coronary artery bypass surgery and offer them appropriate management strategies to improve sleep and reduce anxiety and depression.

## Introduction

Heart disease is the second leading cause of death in Taiwan [1] and half of all deaths are due to coronary artery disease. One of the major therapies to manage severe coronary artery disease is coronary artery bypass grafting (CABG), which accounts for 70% of cardiac surgery in adults in Taiwan [2]. CABG can effectively relieve a patient’s previous symptoms of coronary disease such as discomfort from chest pain. However, recovery from surgery involves wound healing, pain at the surgical sites, and fatigue, which are all stressors that heighten the need for sleep [3]. Obtaining needed sleep is difficult for CABG patients due to postoperative insomnia, somnolence, poor sleep quality, and lack of sleep continuity. Failure to manage sleep problems can affect postoperative recovery and also influence morbidity, mortality and the quality of life [4–9].

Sleep disturbances can occur immediately after CABG and can continue for as long as six weeks after surgery [10–11]. Liou et al. [12] reported that at 1 week post-hospitalization sleep-related issues were among the primary health problems for CABG patients and at 1 month sleep problems ranked second. The percent of patients reported to suffer from sleep disturbances after CABG ranges from 47–68%, depending on the study duration and methods [6, 8, 13]. Although the prevalence of sleep problems is high and can persist for several weeks or even months after acute surgery, for most patients, sleep problems improve continuously with postoperative recovery [7, 13–14].

Changes in sleep patterns after CABG are related to several factors: individual (e.g., age, gender), physiological (e.g., primary sleep disorders, cardiac function, pain, dyspnea, fatigue, nocturia), psychological (e.g., emotional) and environmental (e.g., hospital environment, patient care activities) [5, 7, 15–16]. These factors affect sleep patterns early after surgery, during hospitalization and during the recovery period. Pre-existing physiological factors are predictors of post-surgical sleep quality; patients with primary sleeping disorders [16], or greater cardiac dysfunction prior to surgery [14, 16] are reported to have more sleep disturbances. Following hospital discharge, patients with physical symptoms of dyspnea and nocturia continue to experience disrupted sleep [8, 11, 15]. Redeker and Hedges [5] proposed a multifactorial model for studying sleep patterns after cardiac surgery, which included endogenous influences, environmental/ situational influences, previous sleep disorders, cardiovascular disease, and emotional distress. Postoperative emotional distress frequently occurs after cardiac surgery, which often presents as anxiety and depression and may result from pain, concerns about surgical complications and postoperative lifestyle changes [17–24]. Sleep fragmentation and difficulty falling asleep can further increase the emotional distress and may reduce the patient’s postoperative adaptive capacity [25]. Not only physiological factors but also the psychological factors of emotional distress affect postoperative sleep quality.

Sleep and rest are essential health needs for patients after CABG [26] and sleep quality and emotional well-being have been shown to be positively correlated [6]. Better sleep quality could therefore improve emotional well-being following CABG. Several studies have explored methods for improving sleep quality in other populations: cognitive therapy [27–29] and exercise [30] have been demonstrated to significantly reduce sleep problems in older adults. However, there have been few investigations of sleep management strategies for patients after cardiac surgery [7].

Most studies of patients receiving CABG have focused on postoperative quality of life or physical, psychological or social adaptive distress; studies have not focused specifically on sleep quality, and the role it plays in emotional distress. Advances in cardiac surgery led to implementation of clinical pathways for diagnosis and treatment of cardiac patients with CABG in Taiwan in 2010. This is a cost-effective practice; however the average length of stay has become progressively shorter. Patients are discharged even though they are not fully recovered. Shorter lengths of stay place patients at greater risk of emotional stress once they return home.

Therefore, the purpose of this study was to understand sleep quality and its changes for patients in Taiwan who had undergone CABG 1 week and 1 month following discharge from the hospital; and to investigate the relationship between sleep quality and the emotions of distress, anxiety and depression. Better understanding of changes in sleep quality for patients after CABG surgery will provide nurses and healthcare providers with knowledge that can be used to implement sleep management strategies for these patients during hospitalization and following hospital discharge.

## Methods

### Design

A descriptive correlational design was employed using demographic data and structured questionnaires to determine the quality of sleep and emotional state of CABG patients 1 week and 1 month after hospital discharge.

### Participants and setting

Patients were purposefully selected from a medical center in northern Taiwan and were included in the study by these criteria: ≥ 20 years old, fully conscious, able to communicate in Mandarin or Taiwanese, and had CABG surgery. Patients were excluded if they had psychiatric diagnoses, such as alcohol or medication addiction, or sleep disorders such as sleep apnea, before CABG surgery. Patients meeting the criteria were recruited at their first follow-up visit to the clinic.

### Measurement instruments

#### Demographic and clinical characteristics

Patients’ medical records provided demographic information on patient characteristics (e.g., age, gender, material status), cardiac disease risk factors (e.g., smoking, drinking) and disease related characteristics (e.g., comorbidity, length of hospital stay, medication use and stage of heart disease, which was based on the New York Heart Association (NYHA) Functional Class).

#### Measurement of sleep quality

The Pittsburgh Sleep Quality Index (PSQI) [31] is a self-administered structured questionnaire that measures quality of sleep. Nineteen items generate seven “component” scores specific to sleep quality: subjective sleep quality, latency, duration, efficiency, disturbances, use of sleeping medications and daytime dysfunction. Each component is scored with a 4-point Likert-type scale response, ranging from 0 to 3: the total for the seven components of the PSQI generates a global sleep-quality score, ranging 0 to 21. The global PSQI score is categorized as follows: 0–4 = good, 5–10 = poor, over 10 = presence of a sleep disorder [31]. The PSQI has excellent internal reliability (Cronbach's α = 0.80–0.85) [18, 31–32–]. We used a Chinese version of this questionnaire which has been translated and validated for use in Taiwan [33]. In this study, Cronbach's α for the PSQI after CABG was 0.76 at 1 week and 0.82 at 1 month post hospitalization, indicating an acceptable level of internal consistency.

#### Measurement of emotional distress

The Hospital Anxiety and Depression Scale (HADS) [34] evaluated emotional distress. This self-rating structured questionnaire consists of 14 items in two subscales: HADS-A measures anxiety (seven items) and HADS-D measures depression (seven items). For this study, we defined emotional distress as the total HADS score, which is the combination of HADS-A and HADS-D. Each item of the HADS is scored with a 4-point Likert scale from 0 to 3. Scores for each subscale are constructed by summation, ranging 0 to 21, whereby increasing scores indicate increasing burden. Each subscore is categorized as follows: normal (0–7), mild (8–10), moderate to severe (≥11) [34]. Our study used the Chinese version of the HADS, which was translated and validated by Chen et al. [35]. Validation showed good internal reliability (Cronbach's α = 0.77–0.82) and test-retest reliability (*r* = 0.64–0.83). In this study, the Cronbach's α for HADS-A after CABG was 0.81 at 1 week and 0.80 at 1 month post-hospitalization; the Cronbach's α for HADS-D after CABG was 0.72 at 1 week and 0.79 at 1 month post-hospitalization, indicating an acceptable level of internal consistency for both subscales.

### Data collection

Data were collected at an outpatient cardiovascular clinic of a medical center in northern Taiwan from July 2010 to July 2011. If patients met the criteria for inclusion, they were recruited at the first follow-up visit to the clinic (1 week after hospital discharge). After obtaining written consent, the participants answered the PSQI and HADS questionnaires by self-reporting. Participants answered the structured questionnaires at 1 week and again at 1 month after discharge from the hospital. Demographic and disease-related information were collected from patients’ medical records with their consent.

### Data analysis

Descriptive statistics described the participants. Inferential statistics such as Pearson product-moment correlation, paired t-test, and ANOVA test with Dunnett’s post hoc comparison analyzed the level of sleep quality and its correlated factors for the CABG patients at 1 week and 1 month after hospital discharge. Multiple regression analysis was performed to find the potential predictors of sleep quality after controlling for the variables of demographic and clinical characteristics. All statistical analyses were performed using the SPSS software package for Windows, version 17.0 (Chicago, IL, USA). All *P*-values were two-tailed and data were considered significant at a level of α = .05.

### Ethical considerations

This study was reviewed and approved by the Research Ethics Committee (No. 099-05-122) of Tri-Service General Hospital. The researcher explained the study purposes and procedures in detail and obtained written informed consent from each patient. The patients were assured that they were free to withdraw during the study without any compromise in subsequent treatment. Strict confidentiality was maintained throughout the process of data collection and analysis.

## Results

### Demographic and clinical characteristics

Table 1 presents personal and health-related characteristics of the study sample. The mean age of the 87 participants was 65.2 ± 12.1 years (range 35–89); 65 were male (74.7%) and 22 were female (25.3%). The majority of participants were married, non-smokers and non-drinkers. The number of comorbidities was 3.6 ± 1.6 (range = 1–8); mean length of hospital stay was 21.4 ± 11.1 days (range = 10–81). Emergent CABG surgery occurred in 52.9% of the sample, 81.9% had on-pump cardiopulmonary bypass, and the number of graphs was 2.7 ± 0.8 (range = 1–4). On average subjects were prescribed 13 medications 1 week post-hospitalization and 9 medications 1 month post-hospitalization. At both time points, most of the medications were cardiovascular, diuretics, anticoagulants or antacids. At 1 week post-hospitalization most participants were NYHA class II (n = 55, 63.2%) or III (n = 19, 21.8%); at 1 month post-hospitalization most were NYHA class I (n = 55, 63.2%) or II (n = 29, 33.3%). McNemar’s test confirmed the study sample had a significant improvement in NYHA class from 1 week to 1 month post-hospitalization (*p* < .01).

**Table 1:** Participant demographics (*n* = 87).

Variable	No. (%)	Mean ± SD	Range
**Age (year)**		65.2 ± 12.1	35–89
**Gender**			
Male	65 (74.7)		
Female	22 (25.3)		
**Marital status**			
Yes	66 (75.9)		
No	21 (24.1)		
**Caregivers**			
Yes	80 (82.0)		
No	7 (8.0)		
**Education**			
None	8 (9.2)		
Elementary school	34 (39.1)		
Junior high school	9 (10.3)		
Senior high school	21 (24.1)		
≥ College	15 (17.2)		
**Religion**			
Yes	73 (83.9)		
No	14 (16.1)		
**Smoking**			
Yes	23 (26.4)		
No	64(73.6)		
**Drinking**			
Yes	11 (12.6)		
No	76 (87.4)		
**Number of comorbidities**		3.6 ± 1.6	1–8
**Procedural status**			
Elective CABG	41 (47.1)		
Emergent CABG	46 (52.9)		
**Cardiopulmonary bypass**			
On-pump	71 (81.9)		
Off-pump	16 (18.4)		
**Total number of grafts**		2.7 ± 0.8	1–4
**Length of hospital stay**		21.4 ± 11.1	10–81
**Number of medications**			
1 week post-hospitalization		12.94 ± 3.14	5–19
1 month post-hospitalization		8.92 ± 2.92	2–15
Extent of Heart Failure*			
1week post hospital discharge			
Stage I	13 (14.9)		
Stage II	55 (63.2)		
Stage III	19 (21.8)		
1 month post hospital discharge			
Stage I	55 (63.2)		
Stage II	29 (33.3)		
Stage III	3 (3.4)		

*New York Heart Association (NYHA) Functional Classification.

### Sleep quality post-hospitalization

One week post-hospitalization, average sleep duration for participants was 5.76 hours, and sleep efficiency (total sleep duration ÷ time in bed) was 62.98%. One month post-hospitalization, average sleep duration for participants was 6.36 hours and sleep efficiency was 71.56%, an improvement from 1 week post-hospitalization. Sleep quality was assessed with the PSQI at 1 week and 1 month post-hospitalization (Table 2); the global PSQI score measured overall sleep quality and component scores measured factors relating to sleep quality. At 1 week the mean global PSQI score was 12.39 ± 5.26 points, a level indicative of a sleep disorder. Sleep medication was the lowest scoring component of the PSQI (0.92 ± 1.36) and the highest was daytime dysfunction (2.36 ± 0.90). When participants answered the PSQI at 1 month post-hospitalization, the mean global PSQI score was 9.09 ± 5.12 points, indicating that sleep quality had improved since 1 week post-hospitalization, but remained poor. Sleep medication use remained the lowest component score (0.66 ± 1.11) at 1 month. The highest component score at 1 month shifted from daytime dysfunction to sleep efficiency (1.67 ± 1.13), suggesting this component had the greatest influence on poor sleep quality at 1 month. A paired t-test confirmed that all seven components of the PSQI as well as the global PSQI score had significantly improved between 1-week and 1-month post hospitalization (Table 2). These results indicate that although measures of sleep quality had improved between 1week and 1 month, normal sleep quality had not been attained at 1 month post-hospitalization.

**Table 2:** PSQI scores: 1 week and 1 month post-hospitalization; comparison with paired t-test (*n* = 87).

Component	1-week		1-month			
	Post-hospitalization		Post-hospitalization		Paired	
	M	SD	M	SD	t-test	*p*-Value
**Subjective sleep quality**	2.05	0.95	1.38	0.96	5.52	< .001
**Sleep latency**	1.90	1.16	1.43	1.19	3.93	< .001
**Sleep duration**	1.74	1.27	1.14	1.14	5.55	< .001
**Sleep efficiency**	2.07	1.15	1.67	1.13	3.54	< .001
**Sleep disturbance**	1.44	0.56	1.18	0.42	4.25	< .001
**Daytime dysfunction**	2.36	0.90	1.64	0.90	6.23	< .001
**Sleep medication use**	0.92	1.36	0.66	1.11	2.37	.02
**Global PSQI score**	12.39	5.26	9.09	5.12	6.39	< .001

PSQI: Pittsburgh Sleep Quality Index; M: mean; SD: standard deviation.

To determine how many participants were experiencing poor sleep quality at the two time points and to determine if there was a significant change in this population, we evaluated the percent of participants with poor (>5) and good (≤ 5) sleep quality. When measured 1 week post-hospitalization, 72 participants (82.8%) had global PSQI scores indicative of poor sleep quality; at 1-month post-hospitalization there were 58 participants (66.7%) with global PSQI scores greater than five. We evaluated if this change in percent of participants was significant and also determined if component PSQI scores for subjective sleep quality, sleep latency, sleep duration and sleep efficiency had significantly changed. The four component scores were evaluated as either poor or good by applying cut-off values (shown in Table 3) as suggested by Lemma et al. [36] and the percent of participants with either poor or good scores for the four components of the global PSQI score was determined. The McNemar test indicated that global PSQI sleep quality was significantly improved at 1 week and 1 month. In addition three of the components improved significantly; the exception was the component of sleep efficiency (*p* = .48) (Table 3). Patients’ subjective feelings regarding causes of sleep disturbance remained the same at both time points: going to the toilet, waking up at night, inability to fall asleep again easily, and coughing (data not shown).

**Table 3:** Percent of participants with poor and good sleep quality post-hospitalization.

Components	1 week	1 month	
	Post-hospitalization	Post-hospitalization	P^a^
	No. (%)	No. (%)	
**Subjective sleep quality**			< .001
Poor	58 (66.7)	32 (36.8)	
Good	29 (33.3)	55 (63.2)	
**Sleep latency**			.02
Poor (time ≥ 31 min)	43 (49.4)	30 (34.5)	
Good (time ≤ 30 min)	44 (50.6)	57 (65.5)	
**Sleep duration**			< .001
Poor (< 6 hr)	51 (58.6)	29 (33.3)	
Good (≥ 6 hr)	36 (41.4)	58 (66.7)	
**Sleep efficiency**			.48
Poor (< 85%)	73 (83.9)	69 (79.3)	
Good (≥ 85%)	14 (16.1)	18 (20.7)	
**PSQI global sleep quality**			< .001
Poor (> 5)	72 (82.8)	58 (66.7)	
Good (≤ 5)	15 (17.2)	29 (33.3)	

PSQI: Pittsburgh Sleep Quality Index;

^a^ = McNemar Test.

### Emotional distress post-hospitalization

The HADS questionnaire measured emotional distress of the participants following hospitalization: combined (total) scores for HADS-A (anxiety) and HADS-D (depression) determined the score for overall emotional distress. HADS Scores are summarized in Table 4. The HADS-A scores at 1 week post-hospitalization indicated the presence of anxiety in 27 participants: mild anxiety in 13 (14.9%) and moderate to severe anxiety in 14 (16.1%). One month post-hospitalization, 10 participants had anxiety: six had mild anxiety (6.9%) and four had moderate to severe (4.6%). The HADS-D scores indicated a majority of the participants were burdened with some level of depression at 1 week post-hospitalization (n = 68, 78.1%): 15 participants had mild depression (17.2%) and 53 had moderate to severe depression (60.9%). One month post-hospitalization, 21 participants had mild depression (24.1%) and 31 had moderate to severe depression (35.6%). Pearson product-moment correlation showed a significant positive correlation between depression and anxiety of the HADS at both at 1 week and 1 month post-hospitalization (1 week post-hospitalization *r* = .40, *p* < .01; 1 month post hospitalization *r* = .55, *p* < .01). Paired samples t-test indicated anxiety, depression and overall emotional distress had significantly improved between 1 week and 1 month post hospitalization, as measured by HADS-A (*p* < .001), HADS-D (*p* < .001) and the HADS Total Score (*p* < .001) (Table 4).

**Table 4:** HADS scores: 1 week and 1 month post-hospitalization and the change in score at 1 month (*n* = 87).

	1 week		1 month			
	Post-hospitalization		Post-hospitalization		Change in Score	
	Mean ± SD	No. (%)	Mean ± SD	No. (%)	T Value^a^	*p*-value
**HADS-A (Anxiety)**	5.62 ± 4.82		3.61 ± 3.66		3.86	< .001
Normal (cut-off ≤ 7)		60(69.0)		77(88.5)		
Mild (cut-off = 8–10)		13(14.9)		6(6.9)		
Moderate/severe (cut-off ≥11)		14(16.1)		4(4.6)		
**HADS-D (Depression)**	11.68 ± 5.02		8.75 ± 4.63		4.39	< .001
Normal (cut-off ≤ 7)		19(21.9)		35(40.3)		
Mild (cut-off = 8–10)		15(17.2)		21(24.1)		
Moderate/severe (cut-off ≥11)		53(60.9)		31(35.6)		
**Total Score for HADS**	17.29 ± 8.23		12.33 ± 7.33		5.09	< .001
Normal (cut-off ≤ 14)		33(37.9)		56(64.4)		
Mild (cut-off = 15–18)		18(0.7)		15(17.2)		
Moderate/severe (cut-off ≥19)		36(41.4)		16(18.4)		

SD: standard deviation; HADS: Hospital Anxiety and Depression Scale;

^a^ = paired t-test.

### Correlates of sleep quality post-hospitalization

To determine if there were any relationships between sleep quality (PSQI scores) and variables of participant characteristics or emotional distress, analysis was performed with Pearson product-moment correlation. At 1 week post-hospitalization, there was a small, but significant positive correlation with the global PSQI score for age (*r* = .23, *p* < .05) and length of hospital stay (*r* = .29, *p* < .01). The correlation was larger for NYHA class (*r* = .40, *p* < .01), anxiety (*r* = .37, *p* < .01) and depression (*r* = .54, *p* < .01). Participants had poorer sleep quality if they were older, had a lengthier hospitalization, had poorer heart function, or had anxiety and depression. At 1 month post-hospitalization a small positive correlation remained for sleep quality and age (*r* = .28, *p* < .01). In addition, there was a positive correlation with NYHA class (*r* = .38, *p* < .01), anxiety (*r* = .54, *p* < .01) and depression (*r* = .74, *p* < .01); in other words, greater age, poorer heart function and more emotional distress (exhibited by anxiety and depression) were associated with poor sleep quality at 1 month post-hospitalization (Table 5).

**Table 5:** Correlations between global PSQI scores and study variables at 1 week and 1 month post-hospitalization (*n* = 87).

Study variables	1-week	1-month
	Post-hospitalization	Post-hospitalization
	Global PSQI scores	Global PSQI scores
**Age**	.23*	.28**
**Total number of grafts**	.20	.01
**Length of hospital stay**	.29*	.14
**Number of comorbidities**	.05	.08
**Number of medications**	-.06	.08
**Extent of Heart Failure** ^**a**^	.40**	.38**
**HADS–Anxiety**	.37**	.54**
**HADS–Depression**	.54**	.74**
**Total HADS score**	.54**	.74**

^a^ = Based on New York Heart Association (NYHA) Functional Classification; PSQI: Pittsburgh Sleep Quality Index; HADS: hospital anxiety and depression scale;

*p < .05,

**p < .01.

We examined the relationship of sleep quality and NYHA functional class using ANOVA (Dunnett’s post hoc test confirmed significance). Participants with NYHA class I had global PSQI scores that were significantly better than participants with NYHA class II or III (1 week post-hospitalization F = 8.92, *p* < .01; 1 month post-hospitalization F = 7.12, *p* < .01); the sleep quality of participants with NYHA class II and those with NYHA class III did not differ significantly from each other (*p* = .30; *p* = .70) at 1 week or 1 month post-hospitalization.

We also compared the differences in global PSQI scores with different levels of anxiety and depression to understand the relationship of sleep quality and emotional distress. The anxiety subscale (HADS-A) showed that the sleep quality of participants with moderate to severe anxiety and those with mild anxiety was significantly poorer than for those without anxiety (1 week post-hospitalization F = 13.90, *p* < .01; 1 month post hospitalization F = 15.19, *p* < .01); the sleep quality of participants with moderate to severe anxiety and those with mild anxiety did not differ significantly from each other (*p* = .82; *p* = .49) at 1 week or 1 month post-hospitalization. The sleep quality of participants with moderate to severe depression was significantly poorer than for those with mild depression; the sleep quality of participants with mild depression was significantly poorer than those with no depression at 1 week and 1 month post-hospitalization (1 week post-hospitalization, F = 13.90, *p* < .01; 1 month post-hospitalization, F = 13.90, *p* < .01).

Association between anxiety and depression and sleep quality (global PSQI scores) were examined with linear multiple regression analysis, controlling for age, gender, and the extent of heart failure. Only the depression score was a significant predictor of sleep quality at 1 week (HADS-A anxiety; β = 0.15, *p* = .16, HADS-D depression; β = 0.40, *p* < .001). However, at 1 month post-hospitalization both anxiety and depression were significant predictors of sleep quality (HADS-A anxiety (β = 0.27, *p* < .01) and HADS-D depression (β = 0.43, *p*< .001) (Table 6).

**Table 6:** Predictors of sleep quality between 1-week and 1-month post-hospitalization*.

Predictor	Standardized	Adjusted	Change in	
	Coefficients (β)	R^2^	R^2^	*p*
**1 week**				
**Post-hospitalization**		0.14		
Depression	0.40		0.12	< .001
Anxiety	0.15		0.02	.16
**1 month**				
Post-hospitalization**		0.14		
Depression	0.43		0.09	< .001
Anxiety	0.27		0.05	< .01

*Controlled variables: age, gender, extent of heart failure based on New York Heart Association Functional Classification;

** added the control variables of sleep quality at 1-week post-hospitalization.

## Discussion

To the best of our knowledge this is the first study to focus specifically on sleep quality and emotional correlates in CABG patients in Taiwan 1 week and 1 month following discharge from the hospital. Our findings demonstrate that 1 month post-hospitalization CABG patients had poor sleep quality, anxiety, depression and emotional distress. Depression was a predictor of sleep quality at 1 week and both anxiety and depression were predictors of poor sleep quality at 1 month post-hospitalization.

### Poor sleep quality post-hospitalization

Sleep problems were common in patients who had received CABG 1 month post-hospital discharge. Previous studies have measured sleep quality in post-surgical CABG patients (i.e., 1 week post-surgery, 4 weeks post-surgery). Our study focused on time-points post-hospitalization, the period after patients have been discharged from the hospital. The average length of hospital stay for the participants in this study was 21 days; therefore, 1 week post-hospitalization is the equivalent of 4 weeks post-surgery and 1 month post-hospitalization is equivalent to 8 weeks post-surgery for previous studies.

One week post-discharge the participants reported an average of 5.76 hours of sleep duration, sleep efficiency was 62.9% and global PSQI scores were12.39 ± 5.26. This is similar to values reported by Redeker et al. [6] for sleep quality: sleep duration of 5.8 hr, sleep efficiency of 71.1% and global PSQI scores of 8.22 at 4 weeks post-hospitalization (the equivalent of 1 week post-hospitalization in this study). One month post-hospitalization the participants in our study had a sleep duration of 6.36 hr, sleep efficiency was 71.56% and the mean global PSQI score was 9.09 ± 5.12. The values reported by Redeker et al. [6] for a similar time-point (measured at 8 weeks post-surgery) were somewhat different: sleep duration was 5.9 hr; sleep efficiency was 74.2%; however, the global PSQI score was 6.82. The global PSQI score in our study was higher (9.09), indicating poorer sleep quality, however, the global score of 6.82 reported by Redeker et al. is still considered to reflect poor sleep quality.

The global and component PSQI scores all improved significantly from 1 week to 1 month post-hospitalization. Daytime dysfunction was the primary cause of poor sleep at 1 week post-hospitalization and the secondary cause at 1 month; the primary cause at 1 month was sleep efficiency. In order to determine how many participants experienced improvements in sleep quality we applied cut-offs to scores for the global PSQI and four components of the PSQI and classified these sleep qualities with binary values of poor or good. Although the majority had poor global PSQI scores at 1 week post-hospitalization (82.8%), a significant reduction was seen at 1 month (66.7%). Interestingly, classifying component scores as either poor or good resulted in only three components showing significant improvement between 1 week and 1 month: subjective sleep quality, sleep latency, and sleep duration; the exception was sleep efficiency. Therefore, determining how many participants have poor or good sleep components may be a more precise way of evaluating the group of CABG patients. Sleep efficiency was the second highest component score at 1-week after discharge and the highest at 1-month after discharge and the mean overall score for sleep efficiency improved significantly (2.07 ± 1.15 versus 1.67 ± 1.13). However, when component scores were categorized as either poor or good, the percent of participants with poor sleep efficiency was not significantly lower at 1 month (83.9% versus 79.3%).

Sleep efficiency and daytime dysfunction were the highest component scores of the PSQI 1 week and 1 month post-hospital discharge. In Taiwan, patients believe that bed rest is beneficial to postoperative recovery [37]. They believe they can relieve their discomfort and symptoms or prevent deterioration of heart function by avoiding activity and staying in bed. Poor sleep efficiency and daytime dysfunction can result from prolonged bed rest, which may actually make it more difficult for patients to fall asleep. A reduction in bed rest and appropriate exercise after surgery has been demonstrated to increase cardiovascular endurance and improve physical function [38–39]. Thus, it is recommended that nurses educate post-surgical CABG patients regarding the need for exercise and instruct them on how to implement appropriate activities. This can help improve recovery of heart function in addition to lessening the incidence of daytime dysfunction and sleep disturbance.

For both time points post-hospitalization the most frequent causes for sleep disturbance were going to the toilet, awakening in the night and not being able to fall asleep again easily, and coughing. A high proportion of our participants were prescribed diuretics, therefore it is not surprising that going to the toilet was reported as the primary cause of sleep disturbance. Teaching patients the timing of taking diuretics, in the morning or before 6:00 p.m., can reduce nocturia and avoid sleep disturbances. The study results show that participants experienced a reduction in sleep problems and sleep quality between 1 week and 1 month post-hospitalization, with significantly fewer sleep problems and significantly better sleep quality. These dynamic improvements in sleep pattern with patient recovery were consistent with the findings from other studies [4, 6, 13, 40].

### Anxiety and depression post-hospitalization

At 1 month post-hospitalization four (4.6%) participants had moderate to severe anxiety and 31 (35.6%) had moderate to severe depression. These results are consistent with those of related studies [16–22] and this emotional distress may be related to the differences between their expectations before surgery and their condition afterward [41]. More realistic expectations can be obtained if healthcare personnel explain the overall postoperative recovery process to patients in detail prior to surgery. Providing this knowledge to patients may help relieve their postoperative emotional distress and, as a consequence, improve their sleep quality. Future studies are suggested to further investigate this topic. The degree of emotional distress of subjects at 1 month post-hospitalization significantly improved from the level of distress seen 1 week post-hospitalization. One possible reason could be the overall improvement of the participants’ health. While both anxiety and depression significantly improved 1 month post-hospitalization, 52 participants still had scores indicating a certain level of depression: 24.1% had mild depression and 35.6% had moderate to severe depression. The significant improvement seen in participants with mild and moderate/severe anxiety may be related to the fact that anxiety can be considered a “temporary” emotional reaction to the trauma of major surgery and the patient’s uncertainty of survival [42]; the stress of surgery and hospitalization is reduced after discharge, which may result in the degree of anxiety gradually diminishing. Depression is an emotion of hopelessness and sorrow [43]. Patients might have depression and emotional distress if their overall physical condition or improvement did not meet their expectations. This study found that overall emotional distress, as measured by the combination of anxiety and depression, as well as anxiety and depression alone were negatively correlated with sleep quality during the month following hospital discharge, results consistent with those of related studies [4, 6, 44].

### Predictors of sleep quality post-hospitalization

The dynamic changes in participants’ sleep quality that occurred as a result of CABG surgery continued post-hospitalization because of the resulting physical and psychological stress. Poor sleep quality is a symptom of many health problems. Anxiety and depression were the independent variables in this study that predicted the sleep quality of the participants; those who had higher levels of either anxiety or depression had poorer sleep quality at 1 week and 1 month post-hospitalization. Depression was the major predictor. This result is consistent with previous studies: depression was the major predictor of sleep quality in caregivers [45] and in patients with obstructive sleep apnea syndrome [46]. The effect of depression on sleep quality can be ameliorated if nurses provide appropriate interventions to help patients manage emotional distress in general, as well as anxiety and depression specifically.

Based on Maslow's hierarchy of human needs, the physiological need for sleep is of primary importance [47]. We hypothesized that anxiety and depression were potential predictors of sleep quality and, therefore, defined sleep quality as an outcome variable. Performing linear multiple regression analysis and controlling for age, gender, and the extent of heart failure confirmed our hypothesis: anxiety and depression were significant predictors of sleep quality at 1-week and 1-month post-hospitalization. This has also been demonstrated in previous studies [46]. Reverse modeling, using linear multiple regression analysis and controlling for age, gender, and the extent of heart failure, showed that sleep quality was a significant predictor of anxiety and depression at 1-week post-hospitalization and1-month post-hospitalization, which has also been reported [48–49]. In order to determine if causal relationships exist between emotional distress, anxiety, depression and quality of sleep will require additional studies.

Recent research indicates that anxiety and depression increase the risk of mortality and morbidity after CABG surgery [50]. Even though there is no evidence to prove the relationship between anxiety, depression and sleep quality, we found most of our participants incurred emotional distress, and emotional distress from anxiety (at 1 month) and depression (at both 1 week and 1 month) were significant predictors of sleep quality in this study. Therefore, we suggest that if emotional distress can be reduced with treatment, sleep disturbances and the risk of mortality and morbidity will decrease after CABG surgery. Currently, emotional distress is managed with pharmacological [50–51] and non-pharmacological methods. Researchers have proposed the use of pharmacological interventions by serotonin-specific reuptake inhibitors (SSRIs) as safe antidepressants for use in cardiac patients. A treatment regime with the SSRI escitalopram has been shown to significantly improve depression when used from the preoperative period to one year after CABG surgery, in addition to improving overall mental health and reducing pain. However, escitalopram had no effect on morbidity and mortality events up to the first year after CABG [51]. Non-pharmacological treatments include cognitive therapy [27–28] and exercise [30]. These have been demonstrated to significantly improve anxiety, depression, negative emotions, and sleep problems. Cognitive therapy has been used to reduce the sleep problems of patients, which significantly improved anxiety and depression, as well as emotional distress [52–54].

Depression and anxiety were significant predictors of sleep quality post-hospitalization, but other factors that may affect depression and anxiety in these patients are unknown. It is suggested that future studies include qualitative interviews to investigate the coping strategies CABG patients use after hospital discharge to deal with anxiety, depression and stress, in addition to identifying the focus of these patients’ concerns. Such research can improve our understanding of the factors affecting sleep quality, which can aid in development of appropriate interventions for such patients.

### Limitations and suggestions

There were several limitations to this study. We did not collect data on the body mass index and sleep patterns of the participants prior to surgery. Body mass index might be associated with sleep patterns; the information of body mass index would be helpful in identifying the potential influence of sleep quality after CABG. Moreover, as the preoperative sleep pattern may affect postoperative sleep patterns and recovery, collecting baseline preoperative sleep patterns would help clarify the overall dynamic changes in sleep. This study followed the sleep patterns of subjects until 1 month post-hospitalization and the high proportion of participants still had PSQI scores indicative of a sleep disturbance. Therefore, we cannot determine how much recovery time is required post-hospitalization to see improvements. We recommend long-term longitudinal studies to characterize the overall changes in sleep quality before and after CABG and during the recovery period. The application of a correlational design in this study did not confirm causal relationships between sleep, anxiety and depression. The cause-and-effect relationship may be verified in future studies with a larger sample. Related factors affecting sleep quality could also be identified objectively. Participants in this study were recruited by purposive sampling. One criterion for inclusion was patients who had received CABG and returned to the outpatient clinic for their first follow-up visit. Therefore, patients physically unable to return to the clinic were excluded, which limited participants to those that were primarily NYHA class I and class II patients, with only a few class III. We did not follow NYHA class III and IV patients who could not return to the clinic. The result was a cohort of subjects with a high degree of homogeneity in heart function. For this reason, the results cannot be generalized to patients with poorer heart function. Future study is recommended to use the time of surgery as the cutoff to investigate preoperative and postoperative sleep quality. The sleep quality of patients receiving CABG could be collected in the ward and the clinic. One additional limitation is that this study did not use any objective measurement tools such as actigraphy (to assess sleep pattern) or 6-minute walk test or handgrip strength to assess physical functioning. Study data might therefore be subject to subjective bias. Use of objective measurement tools in future studies can be analyzed and compared with the subjective data. Objective observation of sleep pattern changes and the related factors after CABG could also be performed.

### Conclusion

The presence of depression in CABG patients was a predictor of poor sleep quality at 1 week following hospital discharge and at 1 month both anxiety and depression were predictors. The advantage of this study was not only the focus on sleep quality, but also the observation of changes that occurred between these two times. We were able to determine what factors changed during a 3-week period. Nurses should be advised to help CABG patients improve sleep quality by actively assessing sleep problems, as soon it is feasible after surgery. The PSQI and the HADS instruments do not reflect a clinical diagnosis of anxiety, depression, and sleep disorders. However, these instruments can evaluate the presence of symptoms of anxiety, depression and sleep disturbances; they are easy and quick to administer to determine the overall postoperative condition of sleep quality, anxiety and depression of CABG patients. Multi-dimensional assessment can confirm which patients are at high risk of developing poor sleep quality. Nurses can actively provide related information for patients who have sleep problems. Corrective measures or referral to a related unit for treatment as well as appropriate health education about sleep can be provided. Risk factors for poor sleep quality should be assessed and corrected. The primary sleep disturbance experienced by CABG patients was getting up during the night to use the toilet; proper instruction for taking diuretics can reduce both nocturia and the resulting sleep disturbance. Suitable cardiopulmonary rehabilitation exercise should be provided as soon as patients can exercise to reduce the effects of prolonged duration of bed rest, increase sleep efficiency, and improve physical functioning; all of which can also have a positive impact on emotional distress. The use of preoperative antidepressant treatment regimens may also help stress reduction for post-surgical CABG patients. Additionally, postoperative relaxation techniques and psychological intervention should also be initiated early, prior to hospital discharge. Communication with the clinical staff or participation in a patient support group can avoid the accumulation of negative emotional distress, which can have a positive effect on sleep quality.

## Supporting Information

S1 Dataset Detailed data of this paper. (XLSX)
